# An *In Vitro* Cord Formation Assay Identifies Unique Vascular Phenotypes Associated with Angiogenic Growth Factors

**DOI:** 10.1371/journal.pone.0106901

**Published:** 2014-09-11

**Authors:** Beverly L. Falcon, Michelle Swearingen, Wendy H. Gough, Linda Lee, Robert Foreman, Mark Uhlik, Jeff C. Hanson, Jonathan A. Lee, Don B. McClure, Sudhakar Chintharlapalli

**Affiliations:** 1 Department of Cancer Angiogenesis, Eli Lilly and Company, Lilly Corporate Center, Indianapolis, Indiana, United States of America; 2 Department of Quantitative Biology, Eli Lilly and Company, Lilly Corporate Center, Indianapolis, Indiana, United States of America; 3 Department of In Vivo Pharmacology, Eli Lilly and Company, Lilly Corporate Center, Indianapolis, Indiana, United States of America; 4 Department of Informatics Capabilities, Eli Lilly and Company, Lilly Corporate Center, Indianapolis, Indiana, United States of America; 5 Department of BioTDR, Eli Lilly and Company, Lilly Corporate Center, Indianapolis, Indiana, United States of America; University of Bari Medical School, Italy

## Abstract

Vascular endothelial growth factor (VEGF) plays a dominant role in angiogenesis. While inhibitors of the VEGF pathway are approved for the treatment of a number of tumor types, the effectiveness is limited and evasive resistance is common. One mechanism of evasive resistance to inhibition of the VEGF pathway is upregulation of other pro-angiogenic factors such as fibroblast growth factor (FGF) and epidermal growth factor (EGF). Numerous *in vitro* assays examine angiogenesis, but many of these assays are performed in media or matrix with multiple growth factors or are driven by VEGF. In order to study angiogenesis driven by other growth factors, we developed a basal medium to use on a co-culture cord formation system of adipose derived stem cells (ADSCs) and endothelial colony forming cells (ECFCs). We found that cord formation driven by different angiogenic factors led to unique phenotypes that could be differentiated and combination studies indicate dominant phenotypes elicited by some growth factors. VEGF-driven cords were highly covered by smooth muscle actin, and bFGF-driven cords had thicker nodes, while EGF-driven cords were highly branched. Multiparametric analysis indicated that when combined EGF has a dominant phenotype. In addition, because this assay system is run in minimal medium, potential proangiogenic molecules can be screened. Using this assay we identified an inhibitor that promoted cord formation, which was translated into *in vivo* tumor models. Together this study illustrates the unique roles of multiple anti-angiogenic agents, which may lead to improvements in therapeutic angiogenesis efforts and better rational for anti-angiogenic therapy.

## Introduction

Angiogenesis, the formation of new blood vessels from existing vessels, is a complex multistep process involving numerous growth factors. These steps include initiation, tip formation and sprouting, migration, proliferation, lumen formation, anastamosis, and maturation [1]. While numerous growth factors have been shown to play a role in the angiogenic process, vascular endothelial growth factor (VEGF) appears to have a dominant role [1].

Inhibitors targeting the VEGF pathway have had some success in the clinic; however, the effects of anti-angiogenic therapy tend to result in transitory improvements measured in months. These treatments result in tumor stasis and shrinkage with some resulting in increased survival. Inevitably, however, the tumors return to growth and progression in many patients. A number of possible evasive resistance pathways have been proposed [2]. One feasible evasive resistance mechanism of anti-angiogenic therapies is the induction of other pro-angiogenic factors to re-establish the tumor vasculature. In fact, profiling of gene expression changes associated with resistance to VEGF inhibitors in xenograft models, showed that EGFR and FGFR pathways were upregulated in the stroma [3]. bFGF has also been shown to drive revascularization in the RIP-Tag2 model after acquiring resistance to anti-VEGFR2 therapy [4]. Targeting VEGF and bFGF with a dual inhibitor, has subsequently been shown to inhibit tumor progression after resistance to VEGF inhibition [4], [5].

Numerous in vitro and in vivo assays have been developed to examine the various steps in the angiogenic process including sprouting and tip formation, migration, differentiation, proliferation, lumen formation, and tube or cord formation [6]. Many of these assays are driven by VEGF or have multiple growth factors in the medium. Little is known about the distinct phenotypes and roles of other angiogenic factors in driving angiogenesis. We have developed a basal medium that allows the characterization of other angiogenic growth factors on cord formation. We found that growth factors such as HGF, EGF, and bFGF can induce cord formation in this system. Interestingly, each of the growth factors induces a unique phenotype that can be differentiated and growth factor combinations indicate dominant growth factor phenotypes. This co-culture system with minimal basal medium also allows for the identification of unique pro-angiogenic drugs or factors and translates into in vivo xenograft models.

## Methods

### ADSC and ECFC co-culture cord formation assay

Human adipose derived stem cells (ADSCs) and endothelial colony forming cells (ECFCs) purchased from Lonza (Allendale, NJ) were cultured as previously described [7]. ADSC and ECFC co-culture assays were performed in basal medium (MCDB-131 medium with 30 µg/mL L-ascorbic acid 2-phosphate, 1 µM dexamethasone, 50 µg/mL tobramycin, 10 µg/mL r-transferrin AF, and 10 µg/mL insulin). ADSCs were plated in 96 well plates at 40–50K cells per well and incubated overnight at 37°C, 5% CO_2_. The next day, the media was removed and 4–5K ECFCs were plated on the ADSC monolayer, incubated at 37°C, 5% CO_2_ for 3–6 hours to allow ECFC attachment before the addition of growth factors and/or inhibitors (2–5X) to achieve the indicated final concentrations. The differences in cell counts reflect differences observed with different cell counters. For validation experiments, a modified assay to increase pericyte association was used whereby 15K ADSCs and 3K ECFCs were plated in a 384-well plate. When indicated cell bound growth factors were removed from the ADSC monolayer by a 60 minute treatment with 500 µg/mL sodium heparin (Sigma) in basal medium prior to ECFC addition. Co-cultures were grown for 3 days, at which time they were fixed, stained, and imaged as described below.

### Growth factors and inhibitors

Multiple doses of growth factors were used to determine the optimal dose to use for subsequent studies (data not shown). Vascular endothelial growth factor (VEGF; R&D Systems) was used at 10–20 ng/mL, hepatocyte growth factor (HGF; R&D Systems) at 100 ng/mL basic fibroblast growth factor (bFGF; Invitrogen) at 50 ng/mL, and epidermal growth factor (EGF; Invitrogen) at 20 ng/mL. To block VEGF and HGF signaling, antibodies to VEGF (Bevacizumab; 10 µg/mL) and HGF (R&D Systems; 10 µg/mL) were used. To examine whether additional growth factors were dependent on each other to induce cords, inhibitors to VEGFR2 (Ramucirumab), bFGF (bFGF antibody; Invitrogen), or EGFR (Gefitinib) were used in basal, VEGF, bFGF, and EGF driven cords. Finally, a TGF-β inhibitor (LY2157299), an inactive control (LY596144), and a multikinase inhibitor (SU11248, Sutent) were tested to examine increases in cord or tumor vessels.

### Fixation, staining, imaging, and quantification of the cords

Following completion of the assay, the ADSC/ECFC co-culture was fixed and permeabilized with either 70% ice cold ethanol or 80% ethanol (which gives equivalent staining; data not shown) for 10–20 minutes. Cells were stained essentially as previously described [7]. Endothelial cells were detected with a sheep anti-CD31 (PECAM-1; Sigma; 1∶200) antibody, smooth muscle actin (SMA) was detected with either a Cy3 conjugated mouse anti-smooth muscle actin (Sigma; 1∶200) or a mouse anti-human SMA antibody (Sigma; 1∶200), and nuclei was detected with Hoechst 33342 (Invitrogen; 1∶1000). Secondary AlexaFluor 488 or 647 conjugated anti-sheep and anti-mouse antibodies were used for detection (Invitrogen; 1∶400).

Cord formation images were capture with either a Cellomics Arrayscan VTI or an Acumen eX3 microplate cytometer. Images taken on the Arrayscan were read at a 5X magnification and the tube formation bio-application was used to detect CD31 staining. Total tube area was calculated from 9 fields for each well with 3–4 wells for each treatment. SMA index was calculated from the intensity of the SMA staining and related to the number of cords as previously described [7]. Tiff images of the entire well were collected with the Acumen eX3. The images were then analyzed with a customized Image Pro algorithm to assess the nuclear area, cord area, and SMA area. In addition, custom built Image J software analysis was used to measure over 100 different descriptors (**Table S1**) to differentiate and assess the different growth factor dependent morphologies. Clustering of the different descriptors were performed in an unsupervised manner as to not place particular meaning on each individual parameter, but rather to utilize these characteristics as unique phenotypes.

### Analysis of growth factors secreted by the cord formation assay

Basal medium, media collected from ADSC monocultures, media collected from ADSC/ECFC co-cultures, and media collected from cocultures treated with VEGF were analyzed for growth factors using Luminex beads according to the manufacturers' protocol (R&D Systems and Millipore).

### Effects of TGF-β inhibition inA549 *in vivo* study

A549 NSCLC tumor cells (5×10^6^ cells) were mixed 1∶1 with Matrigel (BD Biosciences) and injected subcutaneously into the flank of CD1 nu/nu male mice. Tumors were allowed to grow to ∼150 mm^3^ (∼7–10 days) prior to treatment. Animals were orally treated by oral gavage with twice a day (BID) dosing of vehicle, a TGF-β inhibitor (LY2157299; 75 mg/kg), an inactive control (LY596144; 100 mg/kg), or a multi-tyrosine kinase inhibitor targeting VEGF (Sutent, SU11248; 20 mg/kg) for 5 days prior to fixation and staining. Tumors were fixed in Zn Tris, processed, and embedded in paraffin. Four micron sections were cut and stained with a CD31 antibody (PECAM; Pharmingen 550274) and Alexa Fluor 488 conjugated secondary antibodies. Each tumor was imaged at 10× with an inverted fluorescence microscope looking at multiple fields residing completely within the tumor that were well-separated and non-overlapping. The number of fields imaged ranged between 2 to 11 fields and depended upon the size of tumor and amount of non-necrotic tissue present. The following total number of fields was imaged for each group: non-treated (43), LY2157299 (45), LY596144 (31), and Sutent (45). The field data for total area of blood vessels (using CD31) as a ratio of total tissue area (using Hoechst 33342) for individual tumors was averaged to derive the individual tumor vessel density values. The individual tumor values within a group were averaged to derive the group vessel density values expressed with standard errors and P-values. Four to eight animals were analyzed in each treatment group.

### Statistical analysis

Results were expressed as means ±SEM. Statistical differences were analyzed by ANOVA with a Tukey or Dunnett's posthoc test using JMP software.

## Results

### Characterization of growth factors present in the ADSC/ECFC co-culture system

**Figure 1:**
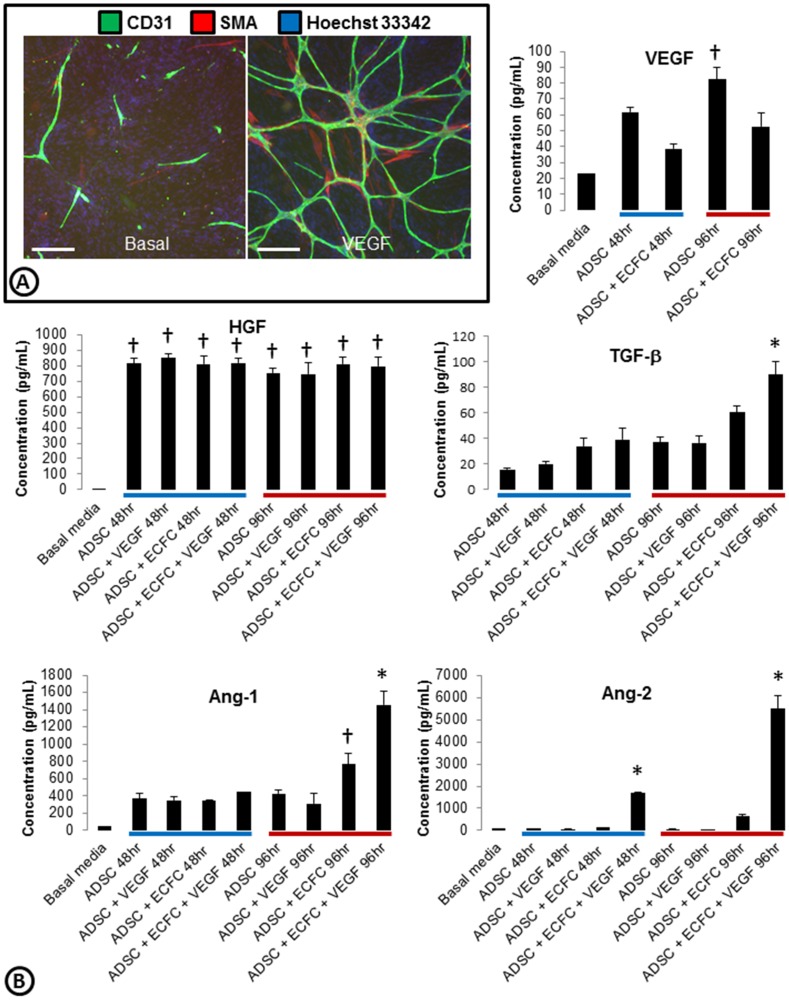
Characterization of growth factors present in the ADSC/ECFC co-culture system. (**A**) Basal or VEGF driven co-cultures of ADSCs and ECFCs were stained for cords (CD31; green), smooth muscle actin (SMA; red), and nuclei (Hoechst 33342; blue). (**B**) Media was collected from ADSCs alone or ADSC/ECFC co-cultures in basal or VEGF driven conditions at 48 (blue bar) and 96 hrs (red bar). Examination of angiogenic growth factors present in the collected media was measured using Luminex. VEGF, HGF, TGF-β, Ang1, and Ang2 had detectable levels above basal media, while bFGF, EGF, and PDGF were not detected (data not shown). n = 3–4 per group with similar results found on two separate experiments. * = p<0.05 vs. all other treatment groups. † = p<0.05 vs. basal media. Scale bars are 250 µm.

Studies indicate that co-cultures of ADSCs and ECFCs lead to robust cord formation with associated SMA positive pericyte-like structures in response to VEGF (**Figure 1A** **, Figure S1**) [7], [8]. This assay can be run in 96 and 384 well format with qualitative analysis on either an ArrayScan or an Acumen ([7] and **Figure S2**). Quantitative validation was performed on a similar assay ran on the Acumen eX3 in a 384 well format (Z′ score  = 0.68 or 0.70 and MSR  = 1.69 or 1.99 for tube area and SMA area analysis respectively; **Figure S2**).

To examine the ability of other angiogenic growth factors to induce cord formation we developed a basal cord formation media which lacks serum and other growth factors typically found in traditional assay systems [7], [9], [10]. To determine which factors were endogenously produced in the ADSC/ECFC co-culture system, Luminex profiling of multiple angiogenic growth factors was examined. Compared to basal medium alone, ADSCs secreted VEGF and HGF into the medium (**Figure 1B**). Co-culturing ADSCs with ECFCs without exogenous growth factors appeared to reduce the levels of VEGF secretion into the medium, but did not alter the secretion of HGF (**Figure 1B**). Interestingly, the addition of VEGF to ADSC monocultures did not change the secretion of any of the factors we examined, however VEGF treatment of the co-cultures increased accumulated secretion of TGF-β and Ang1 at 96hrs and Ang2 at 48 and 96 hours (**Figure 1B**). Additional angiogenic factors such as bFGF, PDGF, and EGF were not secreted above basal levels at any of the time points or culture conditions (data not shown).

**Figure 2:**
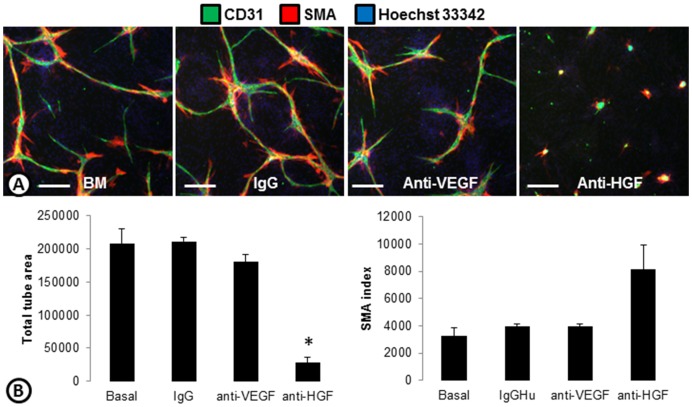
The role of endogenous growth factors in basal cord formation. (**A**) Basal cords made with ADSCs and ECFCs without the addition of growth factors were treated with IgG, or anti-VEGF or anti-HGF antibodies and stained for cords (CD31; green), smooth muscle actin (SMA; red), and nuclei (Hoechst 33342; blue). (**B**) Basal cords treated with IgG, anti-VEGF, or anti-HGF were quantified on the ArrayScan and the total tube area (left) and SMA index (right) are shown. * p<0.0001 vs basal. n = 3 per group. Scale bars are 250 µm.

To confirm the importance of HGF in basal cord formation, antibodies targeting VEGF or HGF were tested (**Figure 2**). Treatment of basal cords with an anti-VEGF antibody did not reduce the cord formation or the associated SMA. Basal cords were significantly reduced, however, when treated with an antibody targeting HGF. SMA was still associated with the few remaining cords causing the SMA index to increase (**Figure 2**).

### Cord formation phenotypes driven by different angiogenic factors

**Figure 3:**
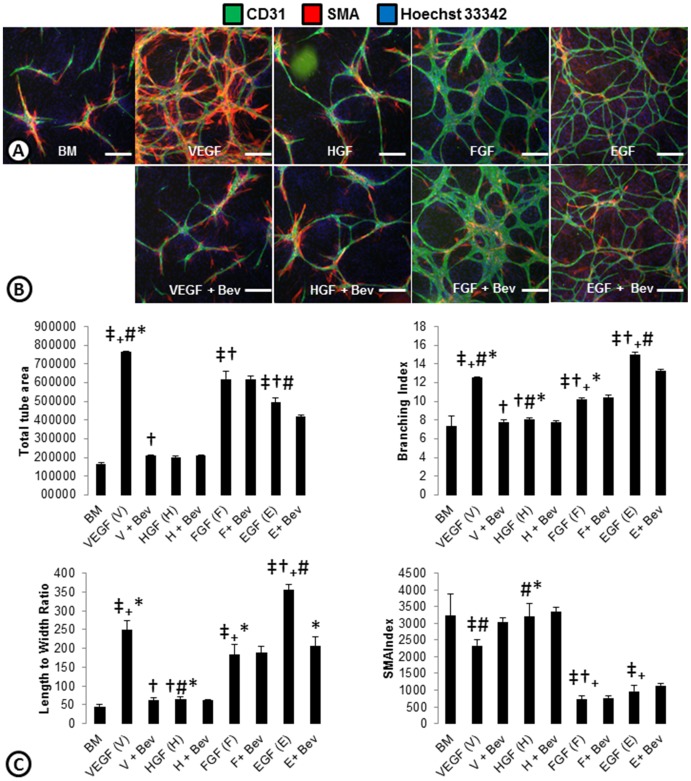
Cord phenotypes driven by angiogenic growth factors. (**A**) ADSC and ECFC co-cultures were stimulated with angiogenic growth factors; VEGF, HGF, bFGF, or EGF and stained for cords (CD31; green), smooth muscle actin (SMA; red), and nuclei (Hoechst 33342; blue). (**B**) To assess whether the cord induction was dependent on VEGF, cords were treated with the different angiogenic factors plus an anti-VEGF antibody (Bevacizumab; Bev). (**C**) ArrayScan quantification of total tube area, branching index, length to width ratio and SMA index are shown as an indication of phenotypes associated with the VEGF, HGF, bFGF, or EGF driven cords. n = 3 per group. ‡ = p<0.05 vs. BM. † = p<0.05 vs. VEGF. # = p<0.05 vs. bFGF. _+_ = p<0.05 vs. HGF. * = p<0.05 vs. EGF. Scale bars are 250 µm.

Most in vitro angiogenesis assays are dependent on VEGF. However, multiple angiogenic factors have been described. Because bFGF and EGF upregulation has been demonstrated in resistance to inhibitors of the VEGF pathway [2]–[5] and are not endogenously present within our co-culture system, we chose to focus our studies on EGF and bFGF driven cords and how they may differ from VEGF and HGF driven cords (**Figure 3**). VEGF stimulation in the ADSC and ECFC co-culture system led to a robust increase in total tube area, a greater branching index, and increased length to width ratio which was inhibited by an anti- VEGF antibody (Bevacizumab; **Figure 3**). Exogenous addition of HGF, which was highly expressed in the basal system (**Figure 1**), did not increase cord formation. If, however, a heparin wash prior to plating ECFCs was performed, which may strip bound-HGF from the basal system, HGF driven cords were seen (**Figure S3**). Exogenous bFGF and EGF, two growth factors that had no detectable expression in the basal system, were able to induce cords in the ADSC/ECFC co-culture system (**Figure 3**). Both of these angiogenic factors increased total tube area, branching, and length to width ratio of the cords; but both had less SMA associated with the cords (**Figure 3**). Qualitatively, bFGF cords appeared to have thicker nodes and EGF thinner nodes compared to VEGF driven cords. Interestingly, while bFGF-driven cords were not affected by Bevacizumab, EGF driven cords had small decreases in some of the readouts (**Figure 3**). Taken together these results indicate that the in vitro basal medium ECFC/ADSC cord formation assay responds to multiple pro-angiogenic factors as observed in tumor models in vivo [4].

**Figure 4:**
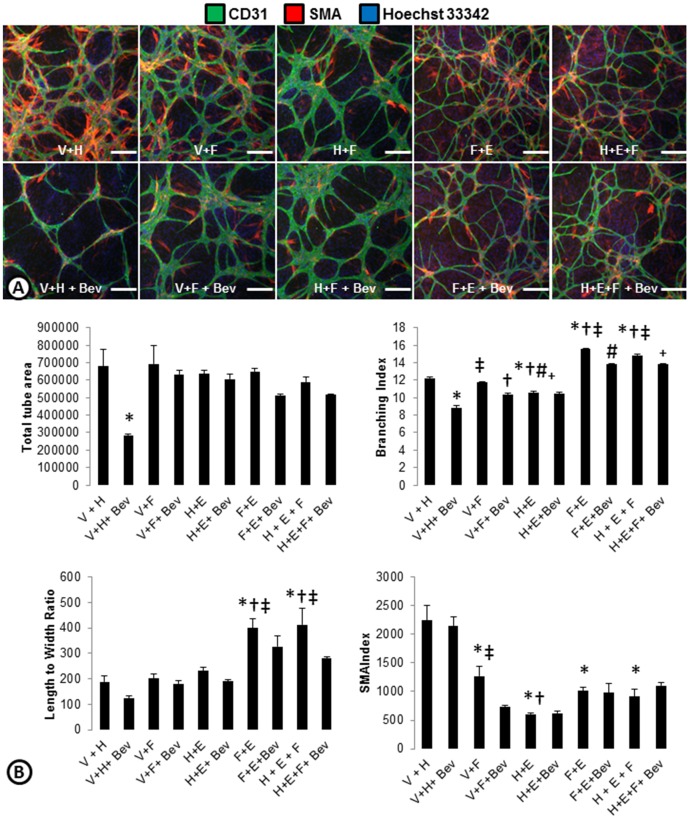
Phenotypes of angiogenic growth factor combinations. (**A**) ADSC and ECFC co-cultures were treated with combinations of angiogenic growth factors (V = VEGF, H = HGF, F = bFGF, E = EGF) with or without anti-VEGF (Bevacizumab; Bev) and stained for cords (CD31; green), smooth muscle actin (SMA; red), and nuclei (Hoechst 33342; blue). (**B**) ArrayScan quantifications of total tube area, branching index, length to width ratio, and SMA index of samples treated with combinations of growth factors with and without bevacizumab. n = 3 per group. * = p<0.05 vs. V+H. † = p<0.05 vs. V+F. ‡ = p<0.05 vs. H+E. # = p<0.05 vs. F+E. _+_ = p<0.05 vs. H+E+F. Scale bars are 250 µm.

**Figure 5:**
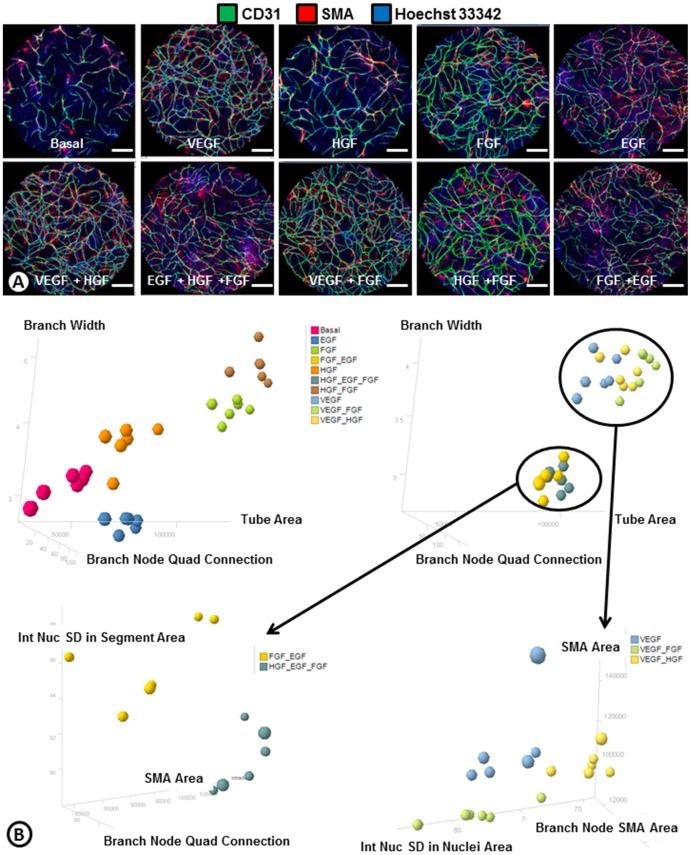
Cluster analysis of phenotypes associated with angiogenic growth factors. (**A**) Whole-well Acumen images of ADSC and ECFC co-cultures treated with angiogenic growth factors or combinations of growth factors following a heparin wash and stained for cords (endothelial cells; CD31; green), smooth muscle actin (SMA; red), and nuclei (Hoechst 33342; blue). (**B**) Cluster analysis based on the top phenotypes from the 101 ImageJ descriptors (Table S1) differentiate the phenotypes of cords driven by angiogenic growth factors or growth factor combinations. The output from statistics comparing the same populations in terms of all the dimensions is shown in Table S2. A p-value less than 0.05 indicate the means of the two groups were significantly different in at least one dimension. Scale bars are 1000 um.

To determine which phenotypes were dominant, growth factor combinations were used to induce cord formation (**Figure 4**). Combining VEGF with HGF led to a phenotype that was similar to the VEGF driven cords (**Figure 4**). Similarly, the combination of bFGF and HGF had cords that phenotypically look like bFGF cords (**Figures 3** **and** **4**). This was likely because (as shown with the single growth factors) the endogenous levels of HGF secreted by the system itself were already high. Interestingly, when VEGF and bFGF were combined the phenotype was almost a hybrid of each growth factor alone (**Figures 3** **and** **4**). These cords were highly branched and had some SMA coverage similar to the VEGF driven cords, but had large nodes indicative of bFGF-driven cords (**Figure 3**). When VEGF was inhibited with Bevacizumab, the phenotype looked more similar to bFGF driven cords (**Figures 3** **and** **4**). When bFGF was combined with EGF, the EGF phenotype appeared dominant as the large nodes and lack of SMA associated with bFGF driven cords was not seen. Instead the cords had some SMA and were long with very small nodes (**Figure 4**). Combining HGF with EGF and bFGF had a phenotype similar to EGF or the bFGF and EGF combo (**Figure 4**). To further understand these vascular phenotypes associated with the different growth factors driving cord formation, an Image J algorithm was developed to measure 101 different endothelial and pericyte descriptors (**Table S1**) from whole well images of the various growth factor driven cords (**Figure 5**). Basal, HGF, bFGF, EGF, and bFGF + HGF driven cord phenotypes were able to differentiate from one another and other treatments using three readouts: branch width, branch node quad connection, and tube area (**Figure 5**). The five remaining growth factor driven phenotypes could then be separated by four additional parameters. bFGF + EGF and HGF+EGF+bFGF were able to be differentiated from the rest based on the nuclear intensity in the segment area, SMA area and branch node quad connection (**Figure 5**). Finally, VEGF, VEGF+bFGF, and VEGF+HGF phenotypes were able to be separated by looking at SMA area, branch node SMA area, and standard deviation of the nuclear intensity in the nuclei area (**Figure 5**). These results also confirm the observations made with the ArrayScan that EGF is the dominant phenotype when combined with bFGF, as the combinations cluster more closely to EGF driven cords. This analysis also indicates that while the addition of HGF to VEGF or bFGF originally didn't show a difference compared to VEGF or bFGF alone, use of a multiparametric analysis can differentiate phenotypes and indicates that the addition of HGF has subtle effects on endothelial-pericyte network phenotypes.

**Figure 6:**
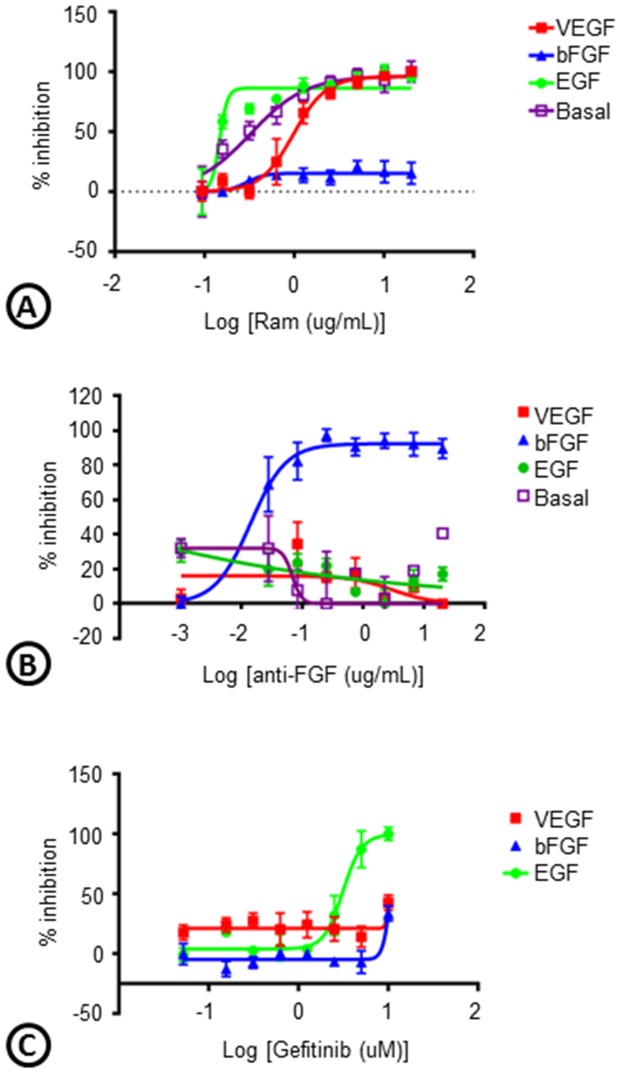
Cross-talk between angiogenic growth factors. Basal, VEGF-, bFGF-, or EGF-driven cords were treated with a VEGFR-2 antibody (IMC-1121B; Ramucirumab; Ram), a bFGF antibody (anti-bFGF), or an EGFR inhibitor (Gefitinib) and the percent inhibition of total tube area from the ArrayScan was graphed. n = 3 per group.

To evaluate whether the phenotypes associated with different growth factor driven cord formation is a direct effect of the growth factors or some secondary signaling, we assessed the effects of inhibition on cord formation. Inhibition of VEGFR-2 with IMC-1121B (Ramucirumab; Ram) blocked basal (IC50 = 0.32 µg/ml), VEGF (IC50 = 0.96 µg/ml), and EGF (IC50 = 0.14 µg/ml) driven cords, but not bFGF driven cords (IC50>10 µg/ml) (**Figure 6**). In contrast, inhibition of cord formation with an anti-bFGF antibody uniquely affected FGF driven cords with (IC50 = 0.013 µg/ml) and a small molecule EGFR inhibitor (Gefitinib) specifically inhibited EGF driven cords (IC50 = 3.1 µM) unless given at high concentrations (**Figure 6**). The IC50 values for anti-bFGF antibody on VEGF and EGF driven cords were greater than 10 µg/ml.

### Pro-angiogenic activity of small molecules

**Figure 7:**
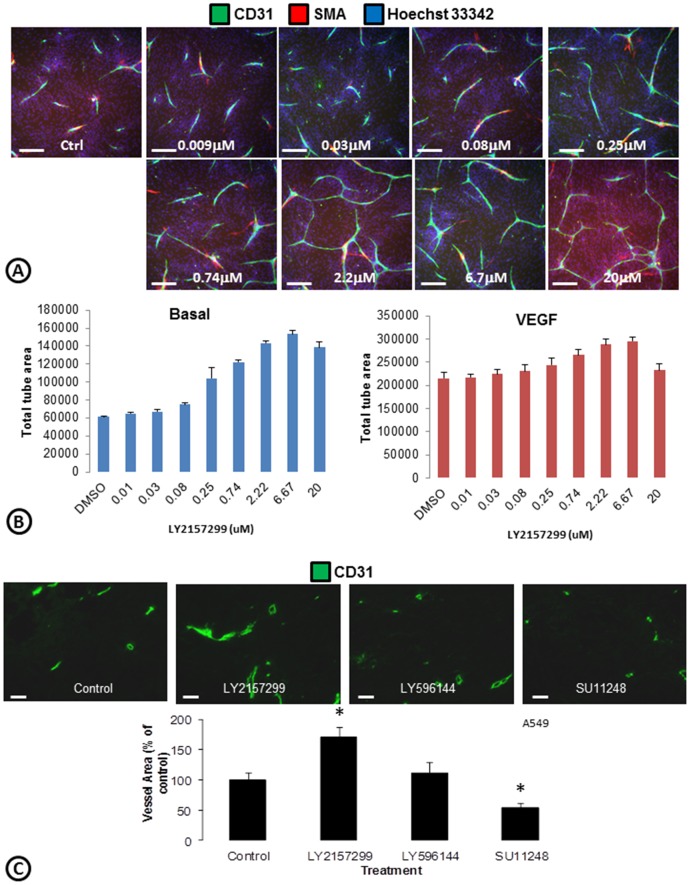
Induction of cord formation with a TGF-β inhibitor. (**A**) Co-cultures of ADSCs and ECFCs were treated with a TGF-β inhibitor (LY2157299) and stained for cords (CD31; green), smooth muscle actin (SMA; red), and nuclei (Hoechst 33342; blue). (**B**) ArrayScan quantifications of total tube area following treatment with LY2157299 in basal medium (left) versus VEGF driven cords (right). n = 6 per group. (**C**) Mice harboring A549 tumor xenografts were treated with a TGF-β inhibitor (LY2157299), an inactive control (LY596144), or sunitinib (Sutent; SU11248) for 5 days. Tumors were fixed, sectioned, stained for tumor vessels (CD31; green), and quantified using Image J. * = p<0.05 vs. control. n = 4–6 per treatment group. Scale bars in A are 250 µm. Scale bars in B are 50 µm.

Development of a medium for the ECFC/ADSC cord formation assay which lacks serum or growth factors allows testing of potential pro-angiogenic small molecules without interference from angiogenic growth factors. It is known that TGF-β inhibits endothelial cell proliferation [11]–[13]. Therefore a TGF-β type 1 receptor inhibitor (LY2157299) was tested in the cord formation assay. LY2157299 increased total tube area by 149% (**Figure 7**) when tested in basal medium but only increased VEGF driven cords by 37%. To assess whether these observations would be translated in vivo, we tested LY2157299 in a xenograft model and observed that the vessel area of A549 tumors significantly increased (**Figure 7C**). In contrast, a structural analog of LY215299 that served as an inactive control compound (LY596144) did not increase vessel area and Sutent (SU11248), a multi-targeted receptor tyrosine kinase targeting VEGFR, decreased the vessel area (**Figure 7C**). A significant increase in tumor vessel area with LY2157299 was also seen in the U87MG glioblastoma tumor xenograft model (data not shown). These results provide a qualitative correlation between these in vitro and in vivo models of angiogenesis, an important aspect if considering an in vitro system for phenotypic drug discovery [14].

## Discussion

Neo-vascularization is a complex process with coordinated cellular signaling between precursor and mature forms of endothelial cells with stromal cells (fibroblasts, smooth muscle cells, and pericytes) coupled with morphological changes including endothelial tube formation, anastomosis, endothelial sprouting, and endothelial vessel stabilization by pericyte recruitment/differentiation. While vascular endothelial growth factor, VEGF, is recognized as the dominant pro-angiogenic factor, other factors such as fibroblast growth factor, epidermal growth factor, hepatocyte growth factor, angiopoietins, thrombomodulin, notch ligands, and transforming growth factor- β are involved as well. Endothelial cord formation in co-cultures of mature endothelial and smooth muscle cells are dependent on addition of VEGF, bFGF, and EGF (16) but do not inform on pericyte differentiation. Development of a basal media co-culture system of progenitor endothelial and stromal cells provide an opportunity to study the roles of multiple angiogenic factors on endothelial cord formation and pericyte differentiation. We found that HGF, bFGF, EGF, and VEGF can all drive cord formation in this system, but each has a unique phenotype that can be differentiated using multiparametric analysis. In general, we find that VEGF has small nodes with many branched and connected tubes and is associated with high levels of SMA. In contrast, bFGF driven cords have thick nodes that are not associated with SMA and EGF has thin nodes with less branching and intermediate levels of SMA. When growth factors were combined, we found that EGF drives a dominate phenotype, while other growth factors have a mixed phenotype when combined (ie VEGF + bFGF). Together these results demonstrate the unique roles of different growth factors in inducing angiogenesis and while different growth factors can induce angiogenesis, the blood vessels that form may not be the same. In addition, we show that use of a basal medium allows one to interrogate these individual roles of the growth factors and allows for the identification of other proangiogenic factors and drugs such as an inhibitor of TGF-β receptor.

Evaluation of growth factors in the ADSC and ECFC co-cultures revealed expression of VEGF, HGF, TGF-β and Ang1 by the feeder layer (ADSCs). Addition of ECFCs decreased the level of VEGF in the medium. This may be a reflection of internalization and degradation of the ligand by endothelial cells [15]. High levels of HGF are secreted by ADSCs and are the main driver of basal cord formation in our system as inhibition of HGF by an antibody almost completely eliminates basal cord formation. Addition of HGF to the system doesn't induce more cord formation, but this is likely due to saturation of the system by bound HGF, as a heparin wash removal of bound HGF prior to addition of HGF leads to significant cord formation. Interestingly, Ang1 is produced to some extent by ADSCs alone, but at the later timepoints there is more Ang1 in the medium when ADSCs and ECFCs are co-cultured. This increase in Ang1 is likely from pericytes associated with the endothelial cells. Ang1 is secreted by pericytes and previous studies have shown that pericyte association with cords began on day 3 and increased with time [7]. Therefore, there are likely only enough pericytes present at the 96 hr time point to effectively increase the Ang1 concentration. Furthermore, recent cell lots of ADSCs that do not produce as much SMA-associated cells do not see a spike even at these later time points (data not shown). Finally, the increase in Ang2 in co-cultures of ADSCs and ECFCs with VEGF stimulation is in alignment with the association between Ang2 and VEGF. Ang2 is secreted by endothelial cells and increases in response to VEGF to induce pericyte dissociation [16]–[18].

bFGF and EGF signaling have been shown to play a role in revascularization of tumors after development of resistance to anti-VEGF/VEGFR2 therapy [3]–[5]. Because these factors were not endogenously present within our co-culture system, we examined the effects of bFGF and EGF driven cord formation. We found that similar to VEGF, both bFGF and EGF can induce cords, but phenotypic differences were observed. These phenotypic differences could reflect differences in mitogenic, sprouting, and survival potencies of the different growth factors. bFGF driven cords were larger in caliber and lacked pericyte coverage. This is consistent with a previous report examining the relationships between VEGF and bFGF in tumor models designed to overexpress both growth factors. Interestingly, pericyte association and maturation was decreased when only bFGF was expressed after inhibition of VEGF expression [19]. Previous reports have also shown that bFGF induced ERK signaling antagonizes TGF-β induced smooth muscle gene expression [20], which may explain the loss of SMA association in bFGF driven cords. EGF driven cords appeared thinner with increased length to width ratios. Based on multiple phenotypic parameters, we were able to differentiate cords driven by individual or multiple factors and were able to establish that cords driven by some growth factors have dominant phenotypes compared to others. This may illustrate an ability to define blood vessels into different classes. Quantitative correlation of the various endothelial-pericyte morphologies with the various growth factors and combinations may lead to predictive biomarkers for inhibiting blood vessels driven by VEGF, bFGF, or EGF.

In addition to bFGF and EGF driving different phenotypic cords, we were able to demonstrate that their ability to induce cord formation is independent of VEGF. Numerous growth factors have been shown to drive angiogenesis, but many of these induce angiogenesis through upregulation of VEGF rather than a direct effect. In our system, we stimulate cord formation with VEGF, bFGF, or EGF but only the VEGF driven cords are dramatically altered by inhibition of VEGF with bevacizumab (a VEGF antibody). Even with growth factor combinations, only the VEGF driven phenotypes appeared to be blocked by the addition of bevacizumab. Similarly, inhibition of bFGF with an antibody only blocked bFGF driven cords. Likewise, an EGFR inhibitor only blocked EGF driven cords. This indicates that these angiogenic growth factors act independent of one another, but can work together to elicit particular angiogenic phenotypes. This also highlights the redundancy in proangiogenic factors and the ability of multiple growth factors to drive angiogenesis. This is particularly important in response to inhibition of a particular pathway, as what has been seen with bFGF driving angiogenesis in response to inhibition of the VEGF pathway [4]. In contrast, we observed some reductions of EGF driven cords with inhibition of the ligand (bevacizumab) or the receptor (ramucirumab). Previous studies have shown that the oncogenic properties of EGFR may be mediated by up-regulation of VEGF, which may explain why we see these effects [21].

In addition to the examination of phenotypic differences between cords driven by various growth factors and the study of dominant growth factor phenotypes in combination treatments, this ADSC/ECFC co-culture system can be used to screen for pro-angiogenic factors or drugs. To address whether this assay system can be used to identify factors that can promote cord formation and potentially be pro-angiogenic, we used a TGF-β inhibitor (LY2157299) in the cord formation assay. TGF-β is known to inhibit endothelial cell proliferation [11]–[13], so one would expect to see increased cord formation with the addition of LY2147299. When LY2157299 was added to VEGF-driven cords or cords in optimized medium, an induction in cords was not apparent. However, when LY2157299 was added into basal medium a significant induction in cord formation was observed. This indicates that some agents that may promote cord formation may be masked in the presence of a milieu of growth factors. Thus, because we have developed a basal medium without exogenously added growth factors, this assay may aid in the identification of new angiogenic factors or drugs that may be useful for driving therapeutic angiogenesis. In addition, the basal medium allows for the identification of other factors that may be combined or inhibited with current anti-angiogenic therapies. This highlights the need to perform these sorts of assays in as minimal of medium as possible to support cord formation without the addition of exogenous angiogenic factors.

In normal epithelial and endothelial cells TGF-β is antiproliferative. However, in tumor cells, parts of the TGF-β signaling pathway are mutated and TGF-β no longer controls the cells. Both tumor and stromal cells have been shown to have increased TGF-β production which is associated with increased tumor invasiveness [11]–[13]. Thus, while in the normal setting TGF-β is anti-proliferative, it has been shown to be an effective anti-tumor target. Despite the increases in tumor angiogenesis we observed with the TGF-β inhibitor, this drug has been shown to elicit anti-tumor effects (Yingling et al. manuscript in preparation). This may reflect differential effects of the inhibitor on endothelial cells versus tumor cells. In addition, previous studies have shown that the number of tumor blood vessels does not necessarily correlate with tumor growth [22]–[25]. Thus, an increase in tumor angiogenesis does not necessarily mean the tumors will grow faster. In fact, this is what we have observed with LY2147299 (Yingling et al. manuscript in preparation).

Despite the evidence that numerous growth factors drive angiogenesis, the majority of the in vitro studies examine a single factor or are performed in media or matrix with numerous growth factors. These studies limit one's ability to understand the different phenotypes driven by the growth factors and how combinations may lead to a particular phenotype versus another. In addition, without the use of a growth factor free medium, many proangiogenic factors may be missed. It would be interesting to relate these phenotypes with function in an in vivo setting to determine whether these phenotypes are also associated with other functional or physiological differences. This may lead to improvements in therapeutic angiogenesis efforts and or better rational for anti-angiogenic therapy in different pathological settings.

## Supporting Information

Figure S1 **Association of SMA-positive cells to endothelial cords.** Co-cultures of ADSCs and ECFCs were stained for cords (CD31) and smooth muscle actin (SMA) and imaged with the ArrayScan. High magnification images show a close association of the SMA-positive pericyte-like structures with the endothelial cords. Scale bars are 250 µm.
(TIF)

Figure S2 **High throughput quantitative validation of the assay.** (**A**) Co-cultures of ADSCs and ECFCs were stained for nuclei (Hoechst), cords (CD31), and smooth muscle actin (SMA) and whole wells were imaged with the ArrayScan. Grayscale images show the image of each marker and the black and white image shows what was analyzed. (**B**) Quantitative validation of Acumen eX3 images was performed in a 384 well assay format. Maximum, minimum and concentration response curves for total tube area and SMA area were used to calculate the overall Z′ and minimum significant ratio (MSR) for each parameter. Scale bars are 250 µm.
(TIF)

Figure S3 **Induction of cords with HGF following heparin wash.** (**A**) To address whether HGF can induce cord formation in the ADSC/ECFC co-culture assay, a heparin wash was performed prior to addition of HGF. After 3 days, the cords were fixed and stained for cords (CD31; green), smooth muscle actin (SMA; red), and nuclei (Hoechst 33342; blue). (**B**) ArrayScan quantification of heparin washed HGF induced total tube area and inhibition with an anti-HGF antibody. * p<0.05 vs basal. n = 3 per group. Scalebars are 250 µm.
(TIF)

Table S1 **101 Image J descriptors.**
(TIF)

Table S2 **Statistical comparison from cluster analysis.**
(TIF)
