# Supplementary figures and images for: An In Vitro Cord Formation Assay Identifies Unique Vascular Phenotypes Associated with Angiogenic Growth Factors
Source: PLoS One. 2014 Sep 11;9(9):e106901. doi: 10.1371/journal.pone.0106901 (PMC4161374; doi:10.1371/journal.pone.0106901)

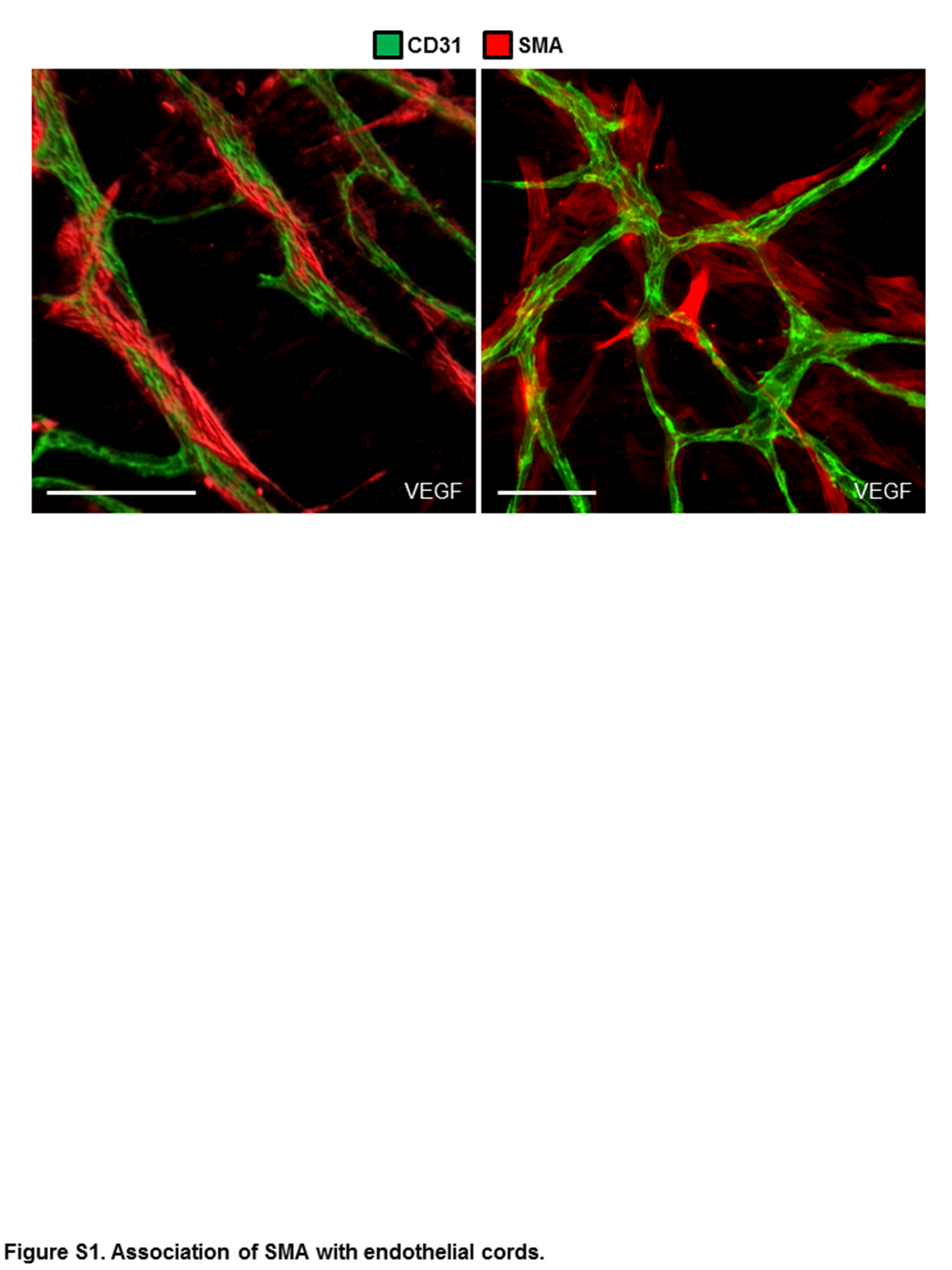

Supplement: Figure S1 — Association of SMA-positive cells to endothelial cords. Co-cultures of ADSCs and ECFCs were stained for cords (CD31) and smooth muscle actin (SMA) and imaged with the ArrayScan. High magnification images show a close association of the SMA-positive pericyte-like structures with the endothelial cords. Scale bars are 250 µm. (TIF) [file pone.0106901.s001.tif]

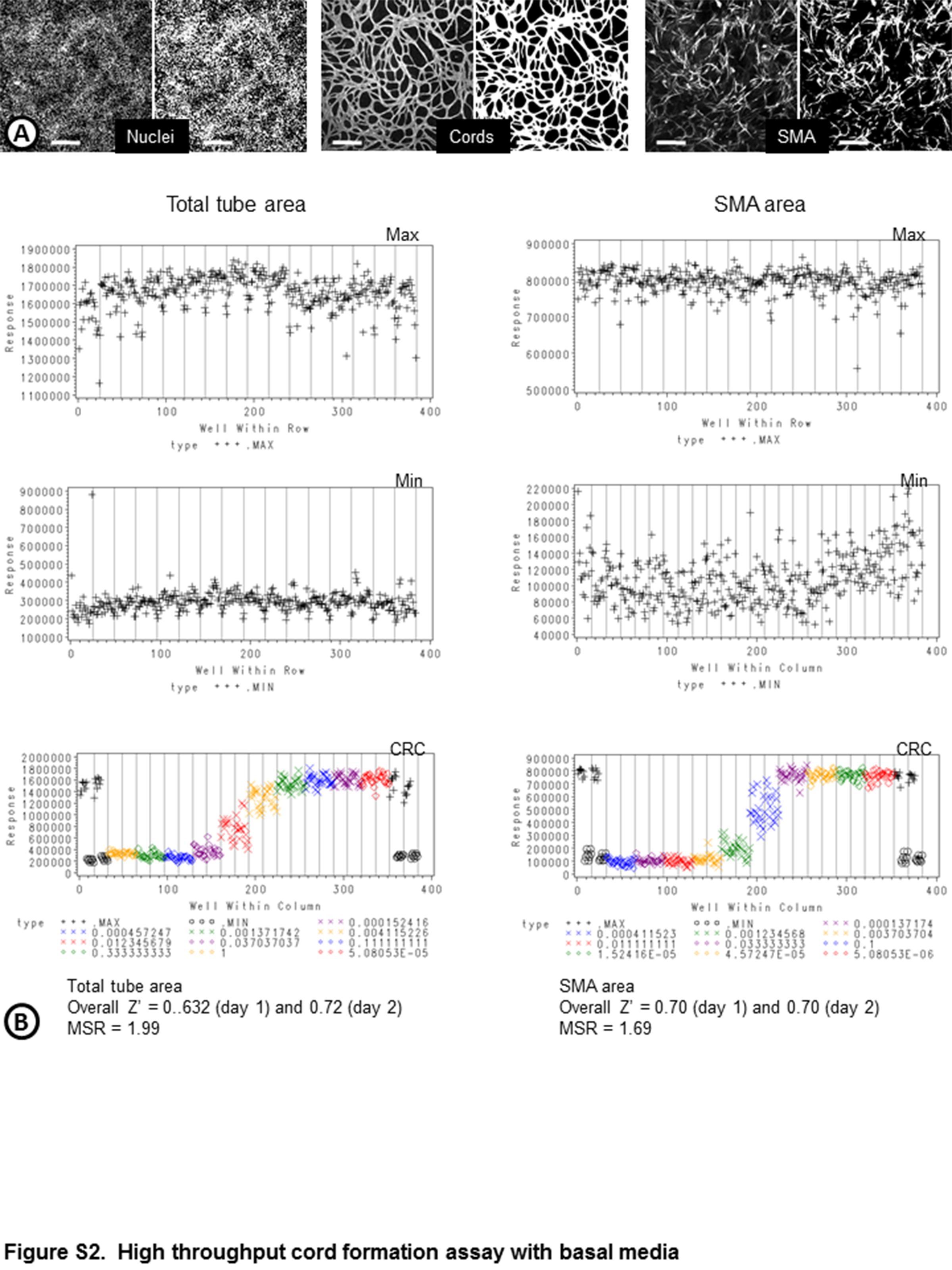

Supplement: Figure S2 — High throughput quantitative validation of the assay. (A) Co-cultures of ADSCs and ECFCs were stained for nuclei (Hoechst), cords (CD31), and smooth muscle actin (SMA) and whole wells were imaged with the ArrayScan. Grayscale images show the image of each marker and the black and white image shows what was analyzed. (B) Quantitative validation of Acumen eX3 images was performed in a 384 well assay format. Maximum, minimum and concentration response curves for total tube area and SMA area were used to calculate the overall Z′ and minimum significant ratio (MSR) for each parameter. Scale bars are 250 µm. (TIF) [file pone.0106901.s002.tif]

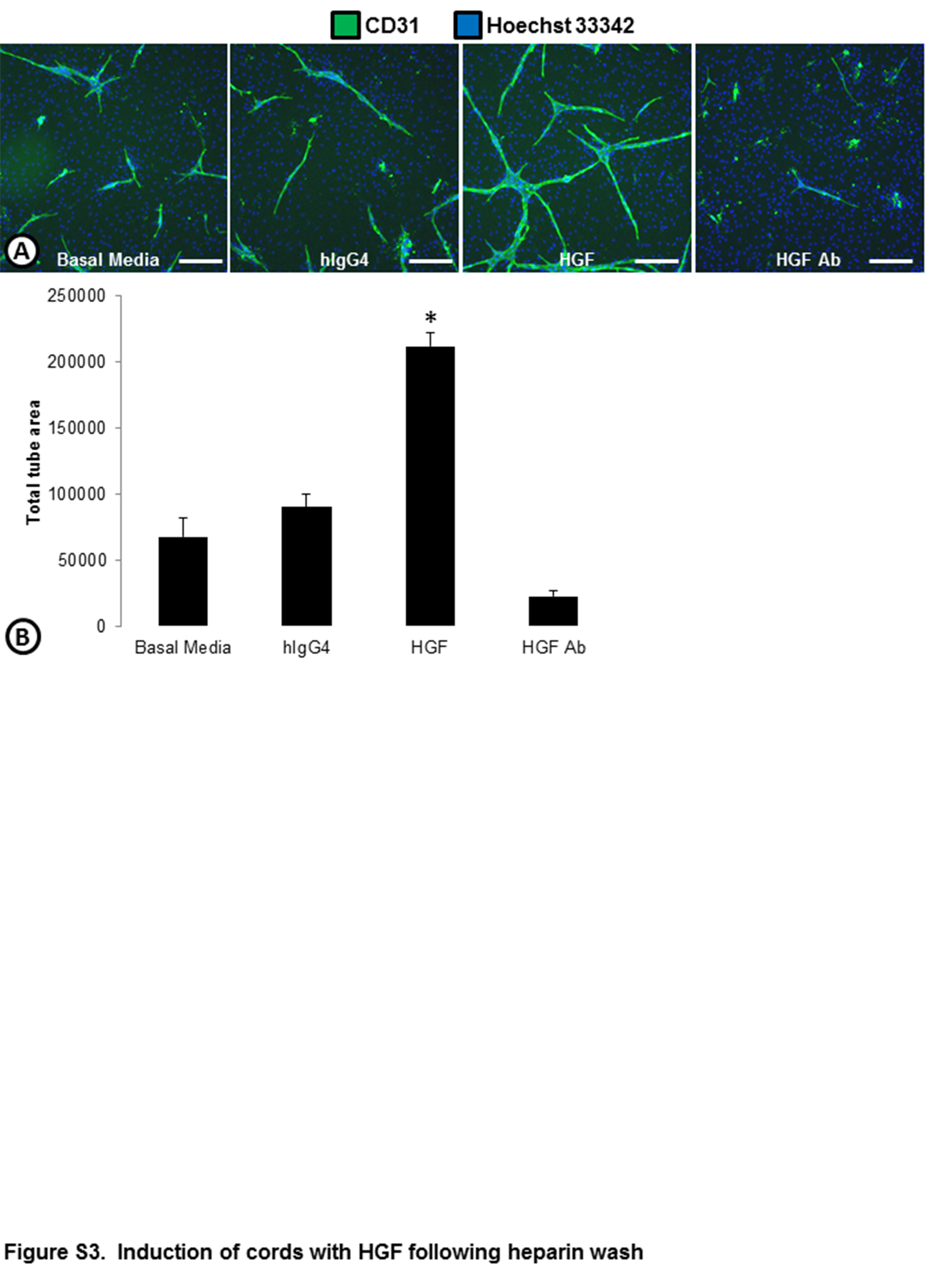

Supplement: Figure S3 — Induction of cords with HGF following heparin wash. (A) To address whether HGF can induce cord formation in the ADSC/ECFC co-culture assay, a heparin wash was performed prior to addition of HGF. After 3 days, the cords were fixed and stained for cords (CD31; green), smooth muscle actin (SMA; red), and nuclei (Hoechst 33342; blue). (B) ArrayScan quantification of heparin washed HGF induced total tube area and inhibition with an anti-HGF antibody. * p<0.05 vs basal. n = 3 per group. Scalebars are 250 µm. (TIF) [file pone.0106901.s003.tif]

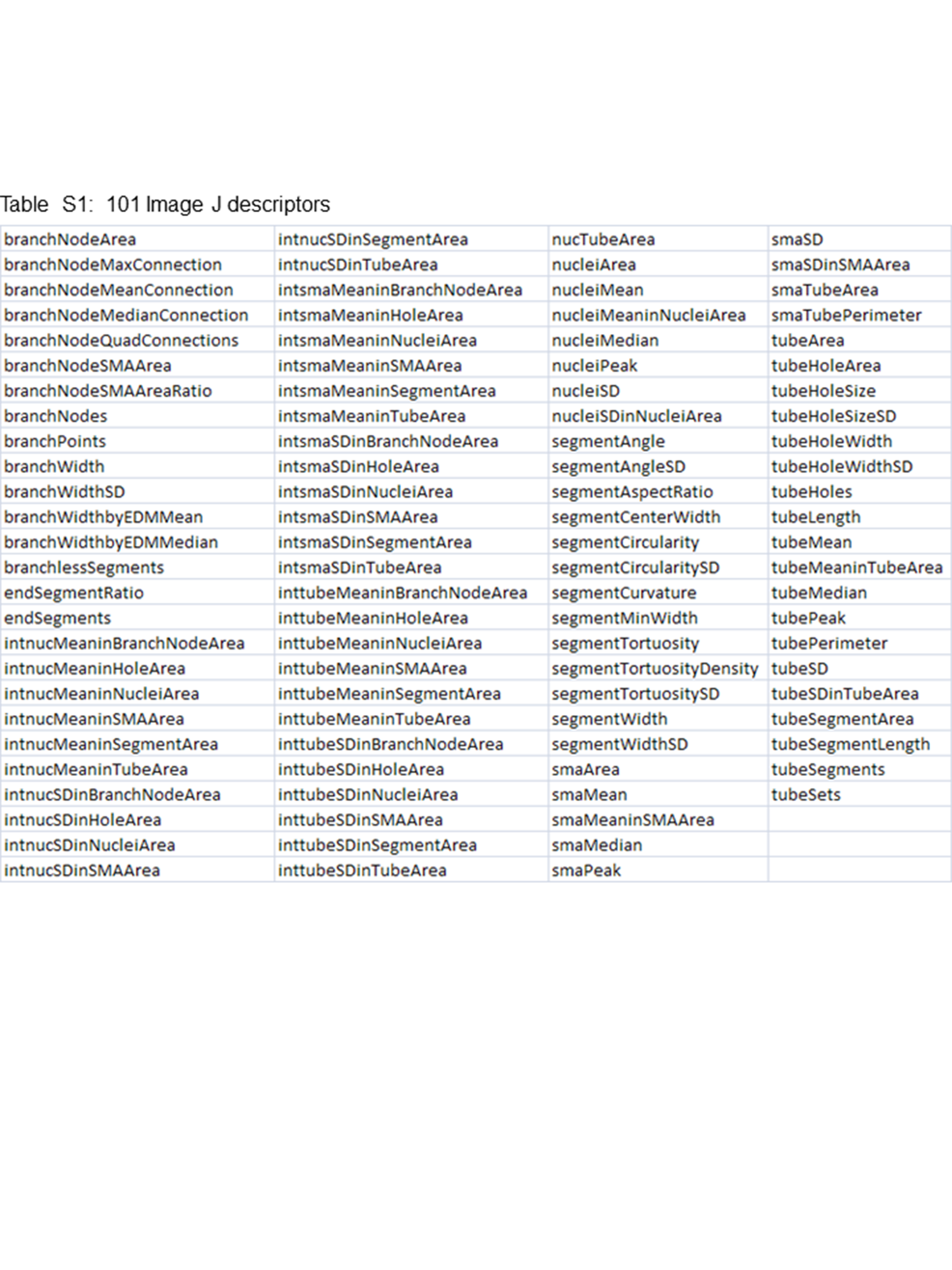

Supplement: Table S1 — 101 Image J descriptors. (TIF) [file pone.0106901.s004.tif]

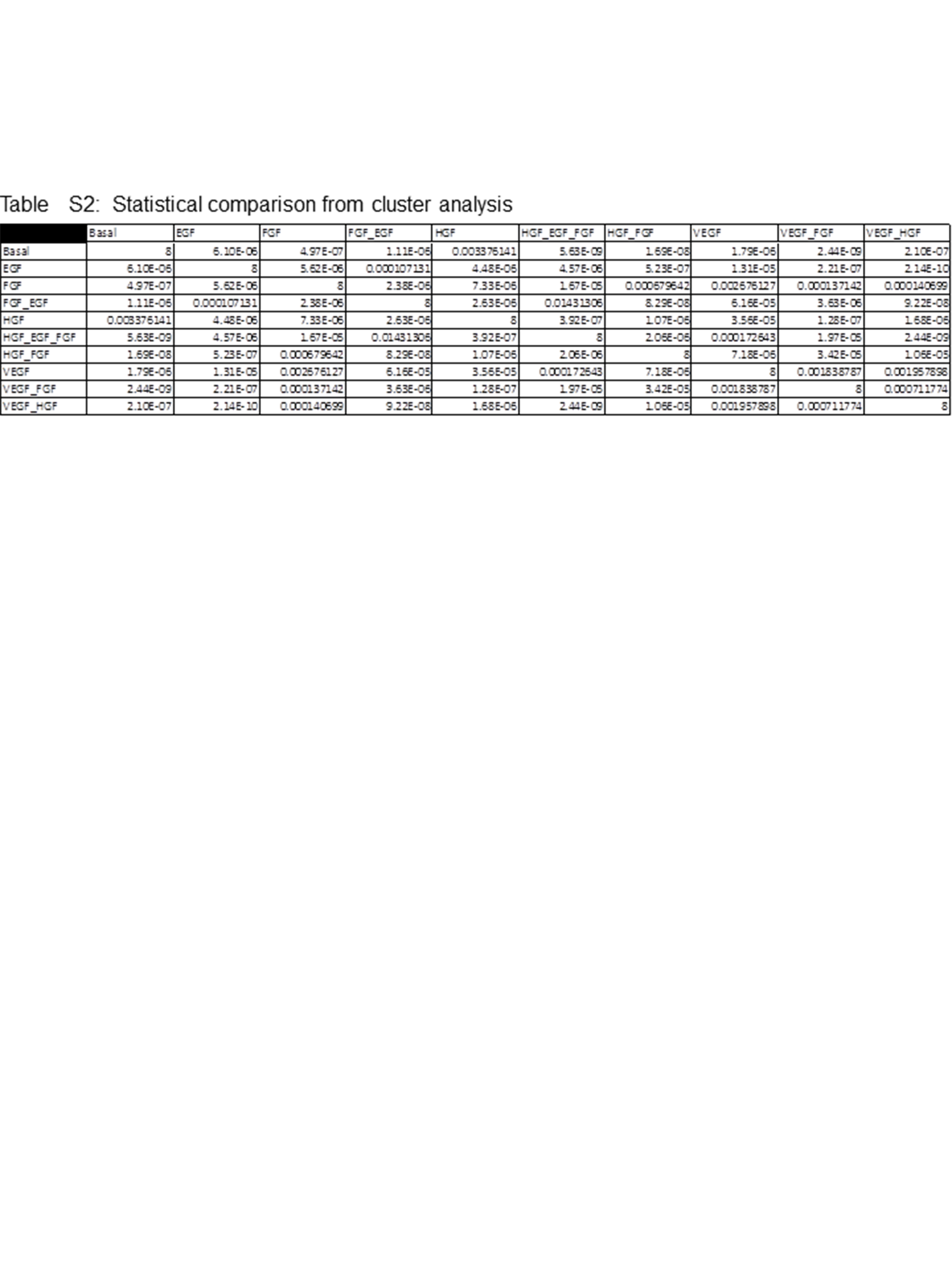

Supplement: Table S2 — Statistical comparison from cluster analysis. (TIF) [file pone.0106901.s005.tif]
